# Bone marrow mesenchymal stem cells and adipocytes in haematological malignancies

**DOI:** 10.3389/or.2025.1704345

**Published:** 2025-11-20

**Authors:** Blanca Gonzalez-Garcia, Cristina Aparicio, Rocio Abia, Francisco J. G. Muriana, Sara M. Jaramillo-Carmona

**Affiliations:** Instituto de la Grasa, The Spanish National Research Council (CSIC), Laboratory of Cellular and Molecular Nutrition, Seville, Spain

**Keywords:** mesenchymal stem cells, adipocytes, dedifferentiation, mitochondrial transfer, bone marrow, haematological malignancies

## Introduction

A range of diseases that affect the blood, bone marrow (BM) and associated organs are classified as haematological malignancies (leukaemia or lymphoma) (1). These pathological conditions often involve the assistance of non-hematopoietic cells in the BM. The progression of haematological malignancies is significantly influenced by BM mesenchymal stem cells (BM-MSCs), a type of non-hematopoietic cell (2). Tumour cells induce deviations in the functional identity of these and other surrounding supporter cells by releasing pro-oncogenic signals in exosomes or as soluble factors, as well as through direct cell-to-cell contact. BM-MSCs can even lead to drug resistance and disease relapse. It can be assumed that the changes induced in BM-MSCs by haematological malignant cells are reversible (referred to here as “touched” cells), or that only the necessary number of BM-MSCs are irreversibly affected (referred to here as “lost cells”) in order for the disease to persist. Conversely, BM-MSCs exhibit a pivotal role in expediting immune system modulation, tissue repair and regeneration, and differentiation into different cell types, including adipocytes, osteoblasts, chondroblasts and nerve cells. Adipocytes and osteoblasts exist in a dynamic equilibrium in BM, which is regulated by an adipogenic differentiation programme that is mutually exclusive with the osteoblastogenic differentiation programme of BM-MSCs (3). They also support the self-renewal and pluripotency of haematopoietic stem cells (HSCs). These properties make BM-MSCs, if they are in a fully healthy state, essential for BM and bone homeostasis (4). The principles of this intriguing behaviour of BM-MSCs are twofold, as they contribute to both the maintenance of BM health gain and the maintenance of BM health loss.

A decline in the quantity of BM-MSCs, in parallel with myelosuppression and damage to the BM vasculature, is associated with the use of some aggressive, clinically adopted therapies to combat haematological malignancies. After this, BM-MSCs are able to repopulate their functional pool (5). The question of how BM-MSCs temporarily escape these lethal BM environments, whether they are chemical, physical or biological, remains unanswered. Interestingly, this exquisite sensitivity of BM-MSCs contrasts with the particular resistance of BM adipocytes, in which BM-MSCs rapidly differentiate at the onset of haematological disorders and during the treatment of established haematological malignancies (6–8). This implies that osteogenesis is reciprocally suppressed and that there are alterations in bone quality and mass (9,10). Signalling pathways such as TGF-β (transforming growth factor β)/BMP (bone morphogenetic protein), WNT (wingless), SHH (sonic hedgehog), NOTCH and FGFs (fibroblast growth factors) are crucial in controlling adipocyte commitment by regulating key transcription factors such as PPARγ (peroxisome proliferator-activated receptor γ) and C/EBPs (CCAAT/enhancer-binding proteins) (11). It is expected that only healthy BM-MSCs differentiate into BM adipocytes, remaining “touched” and “lost” BM-MSCs faithful to the disease. In a recent study, the overrepresentation of blast cells in the BM of adult patients with B lineage acute lymphoblastic leukaemia has been seen to result in the depletion of BM adipocytes, or a significant reduction in their size, due to the failure of BM-MSCs to differentiate (12). The *in vitro* adipogenic capacity of BM-MSCs is also reduced at diagnosis in children affected with the same disease (13). These may be examples of an imbalance transcending temporal considerations between the pool of healthy BM-MSCs and the pool of unhealthy “touched” or “lost” BM-MSCs, which may imply a poor prognosis. All above emphasises that the MSC-adipocyte axis in the BM is a cornerstone of the pathogenesis and treatment of currently incurable haematological malignancies.

## BM adipocytes and their dedifferentiation are key to protecting BM-MSCs

BM adipocytes can revert to their stem cell form through dedifferentiation (14). This suggests an ongoing “you-by-me” exchange between BM cells (the parent and its offspring) to prevent the collapse of the BM induced by disease, and even to promote BM repair. Indeed, the dedifferentiation of BM adipocytes has been shown to contribute to the achievement of a certain degree of homeostasis, similar to that observed in other stem cell systems. A reduction in the size of the stem cell pool can generally result in spontaneous dedifferentiation *in vivo* (15). The process involves the primary transmission of signals from the mitochondria to the nucleus, reporting their metabolic state. Chromatin accessibility (through pioneer transcription factors) and remodelling (through the methylation and demethylation of histone tails) facilitate effective nuclear counterprogramming by enabling transcriptional and epigenetic regulation to be deployed. The decision of BM adipocytes to revert to their multipotent progenitor cells is probably neither autonomous nor straightforward, given that they are constantly receiving interference from haematological malignant cells and instructions to maintain tissue homeostasis from healthy BM cells. The morphology transitions from a rounded state to a spindle-like state, which requires a high and controlled energy supply (sufficient ATP) for the reorganisation of the cytoskeleton and metabolism. This is accompanied by the recovery of the typical mesenchymal immunophenotype and the expression of stem cell genes, thereby restoring the original identity of the BM-MSC. Common features of dedifferentiated cells are regaining of stemness, increased multipotency and resistant to hostile microenvironments (16).

Healthy dedifferentiated BM-MSCs could support other healthy undifferentiated BM-MSCs and rewire unhealthy “touched” BM-MSCs. The challenge lies in controlling the mechanisms, particularly the dedifferentiation of BM adipocytes at the right time, in order to strengthen healthy BM-MSCs against haematological malignant cells and correct the imbalance between healthy BM-MSCs and unhealthy (“touched” and “lost”) BM-MSCs. As there are more adipocytes in the BM, the healthy BM-MSC niche would be enriched by dedifferentiation of BM adipocytes. It is applicable to both in adults and paediatric patients. The difference is that the baseline level of BM adipocytes is expected to be much higher in adults than in children (17,18). In contrast to adults, children have more robust and active MSCs and HSCs in their BM because their bones and haematopoietic systems are still undergoing growth. This may influence the strategy for dedifferentiating BM adipocytes in haematological malignancies, mainly also due to the heterogeneous origin of these diseases (19). In this context, the specific local microenvironment of the BM of an adult or paediatric patient will determine the extent to which adipogenesis progresses in that BM (20). The key to survival may be the level of adipocytes in the BM that are sufficient for dedifferentiation to be successful.

Several strategies for *in vivo* reprogramming of differentiated cells have been documented in preclinical models. These include the use of doxycycline, epigenetic modulators, oncostatin, adenoviruses, paramyxoviruses, synthetic mRNA, and small molecules such as forskolin and dorsomorphin (21). Recently, computational approaches for discovering cocktails of novel molecules that can induce cell reprogramming with high efficiency have been updated (22). *In vitro*, activation of the Wnt/β-catenin, TGF-β/Smad and Notch signalling pathways using Wnt3a, TGF-β1 and FIZZ1, respectively, is known to result in the dedifferentiation of human adipocytes (23). This suggests that genes or pathways involved in adipogenesis play a role in the dedifferentiation of adipocytes. However, further research is needed to develop precise methods for dedifferentiating BM adipocytes and generating healthy MSCs within the BM of patients with haematological malignancies. The Biobanking Working Group of the International Bone Marrow Adiposity Society recently provided an excellent overview of the technical and biological challenges associated with studying BM adipocytes *in vitro* and *ex vivo*, with some of these approaches mimicking the *in vivo* microenvironment (17).

Note that the reprogramming of BM-MSCs towards BM adipocytes and the counterprogramming of differentiated BM-MSCs (dedifferentiation of BM adipocytes) may also be part of a self-protective skill of healthy BM-MSCs, for example to avoid mutations. This is because, while BM-MSCs remain in the form of BM adipocytes, they do not need to undergo long-lasting cycles of clonal expansion to maintain their pool in the BM (asymmetric cell division) or to fulfil regenerative requirements outside the BM (symmetric cell division) (24). In addition, stem cells exhibit a different dynamic pattern to that of differentiated somatic cells over time (25), indicating that BM-MSCs and BM adipocytes may respond to ageing in an asynchronous manner. These pieces of evidence suggest that the process of dedifferentiation in BM adipocytes may serve as a redundancy mechanism, safeguarding the health and youthfulness of the BM-MSC niche. This is especially pertinent in age-associated haematological malignancies (26). The large number of BM adipocytes that accumulate exponentially from birth to old age and are ready to dedifferentiate could therefore be considered of incalculable homeostatic value.

## The property of mitochondrial donation by BM-MSCs

An intriguing difference between BM-MSCs and BM adipocytes is their mitochondrial reorganisation (27). This probably provides high metabolic flexibility, enabling these cells to adapt the ATP-generating pathways in response to changes in the BM environment. BM-MSCs have fragmented mitochondrial morphology, whereas BM adipocytes have large mitochondria due to reduced fission and increased fusion. These dynamic mitochondrial changes make BM adipocytes highly dependent on oxidative phosphorylation. The accumulation of reactive oxygen species and large amounts of ATP is caused as a result, and it is likely that key survival advantages are endowed through stress adaptation, which may at least partly explain the robust resistance of adipocytes in the BM. However, enhanced mitochondrial activity resulting in senescence is observed in BM-MSCs from patients with acute myeloid leukaemia (28). Under additional distressing conditions, such as those triggered by certain types of therapies for treating haematological malignancies, the most substantial impact on BM-MSCs is considerable damage to mitochondria (29). Therefore, when attempting to rescue “touched” BM-MSCs that are metabolically impaired, or to protect dedifferentiated BM-MSCs in haematological malignancies, attention should be given to these organelles. In addition, to recover self-renewal and improve the survival and curative properties, particularly in “touched” BM-MSCs, it is necessary to strike a balance between mitochondrial energy transfer from nutrients and safeguarding carbon skeletons for anabolic reactions (30), including anaplerosis and cataplerosis.

A recently discovered mechanism of intercellular communication, whereby BM-MSCs sustain the metabolism and bioenergetics of other BM cells, which could include their own clonal sisters (healthy or “touched”) and dedifferentiated (healthy) BM-MSCs, is the transfer of mitochondria (31). Interestingly, BM-MSCs are the primary donor cells of healthy mitochondria in the BM. There is no transfer of energy, but of the organelles containing the machinery that can perform this function (including mitochondrial DNA, which can only encode the 13 proteins involved in the electron transport chain that enable cells to respire). This heterotypic phenomenon is akin to a cell-to-cell (autologous) transplant that dramatically increases mitochondrial biomass and competence in the recipient cell, which is then empowered (32). The new cytosol also receives impactful metabolic inputs from the mitochondria and other cellular components, such as vesicles from Golgi and lysosomes, which have senomorphic properties and promote a senolytic phenotype. The process may be initiated when danger-signals are released from damaged mitochondria in unhealthy neighbouring BM-MSCs and enter healthy BM-MSCs, activating mechanisms such as the induction of HO-1 (haem oxygenase-1), AMPK (AMP-activated protein kinase) and PGC-1α (PPARγ co-activator-1α) for increasing mitochondrial biogenesis (33,34). The following step is the vectorised mobility and delivery of healthy mitochondria and additional cellular content through the transient formation of tunnelling nanotube structures between connected cells, which utilise peripheral membrane proteins Connexin-43 and Miro-1/RHOT1 (Rho GTPase 1), most likely with the guidance of the mitochondrial outer membrane protein GAP43 (growth associated protein 43) (35). Other selected ways of mitochondrial cell transfer are exosomes containing mitochondrial-derived vesicles, microvesicles containing entire mitochondria by an outward budding process, free/naked mitochondria and depolarised mitochondria by the pathway of secretory autophagy (36). Remarkably, mitochondrial transfer has the potential to augment the efficacy of contemporary therapeutic immune cell-based therapies, including CAR-T cell therapy (32). In addition, mechanisms involving extracellular vesicles from extramedullary MSCs containing factors related to telomere function and maintenance, such as PCNA (proliferating cell nuclear antigen), has been shown to contribute to the rejuvenation of BM-MSCs in the medullary environment (37).

The transfer of healthy, functional mitochondria from MSCs to recipient cells has been demonstrated *in vitro* and *in vivo*. This extremely powerful biological activity usually occurs spontaneously and endogenously when a cell with dysfunctional and/or depleted mitochondria comes into contact with a healthy cell that is capable of donating mitochondria (38,39), even under conditions of chemotherapy stress (40). Nevertheless, some therapeutic strategies have been recently reported to enhance the mitochondrial transfer. For example, the rejuvenation of senescent BM-MSCs by the transfer of mitochondria from healthy BM-MSCs was observed *in vitro* and *in vivo* after the use and administration of encapsulated melatonin (41). The combination of pioglitazone and iron oxide nanoparticles also enhances the mitochondrial biogenesis and intercellular transfer efficiency of MSCs (42). In certain types of cancer, including haematological malignancies, BM-MSCs can also mediate the mitochondrial transfer to malignant cells (39,43). This would encourage their development and make them resistance to microenvironmental cues, such as survival-promoting cytokines. As with the dedifferentiation of BM adipocytes, mitochondrial transfer is probably only effective in periods of disease inactivity. Further research is therefore needed to develop precise methods of increasing the natural propensity of healthy BM-MSCs to transfer mitochondria to “touched” BM-MSCs within the BM of patients with haematological malignancies at the appropriate time.

## Discussion

**FIGURE 1:**
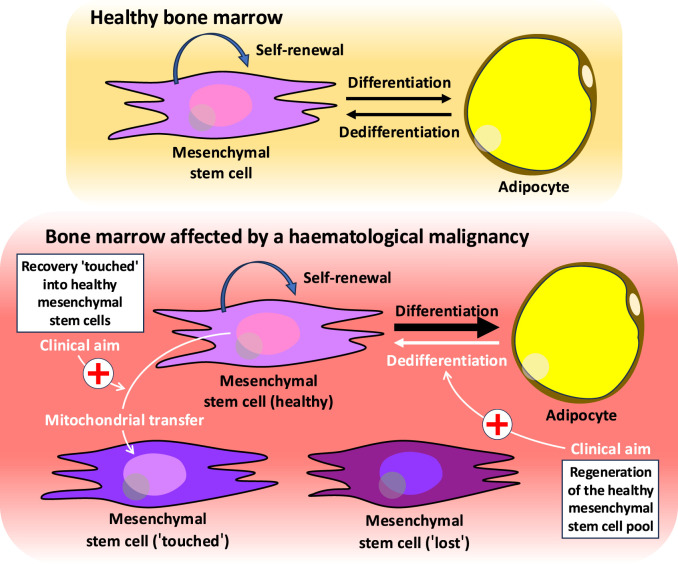
Closing the net on haematological malignancies by dedifferentiation and mitochondrial transfer. A novel therapeutic approach to combating haematological malignancies could be found in the dedifferentiation of bone marrow adipocytes coupled with the transfer of mitochondria from healthy bone marrow mesenchymal stem cells. If “touched” (reversibly dysfunctional) bone marrow mesenchymal stem cells receive a boost of healthy mitochondria, they could regain the strength to make the bone marrow mesenchymal stem cell niche powerful. This could support the healthy bone marrow haematopoietic stem cell niche in conditions such as the remission of the haematological malignancy.

Unravelling the roles of BM-MSC differentiation and BM adipocyte dedifferentiation and the precise regulation of these processes in haematological malignancies holds significant clinical promise for new approaches to the treatment of these diseases. For example, methods could be developed that reverse the orientation of the MSC-adipocyte axis, while also promoting the ability of dedifferentiated BM-MSCs to donate mitochondria and regenerate healthy HSCs when haematological malignant cells survive least within the BM following first-line of targeted therapy. Counterprogramming of BM adipocytes should probably be induced at a convenient time (“dedifferentiation window”) during disease remission (Figure 1). These studies can be successfully translated into clinical practice by using preclinical systems that reflect human BM to some extent, as these systems are unlikely to fully recapitulate all aspects of BM biology. One example of this is BM organoids, which are a cutting-edge model that can be used to examine the behaviour of BM cells, including both haematopoietic and non-haematopoietic cells (44). The flexibility of an organoid is advantageous because it can be customised with cells from each patient, whether adult or paediatric, thereby producing personalised results.

The sudden replenishment of the BM-MSC niche with healthy, dedifferentiated BM-MSCs has the potential to engender a robust and healthy HSC niche, which could dislodge malignant cells from their concealed locations into milieus where they become more vulnerable to subsequent waves of targeted therapy. This reshaping of ecological competition could pave the way for haematological malignant cells to become unable to thrive, thereby helping to eradicate minimal residual disease. In addition, its operational utility could be addressed following myeloablation to prevent relapse. These proposed, multi-layered strategies for tailoring combination therapies to regenerate BM affected by haematological malignancies may inspire new hypotheses and areas for further investigation. The main objective is to improve patient outcomes and advance oncology research.
